# International retrospective natural history study of *LMNA*-related congenital muscular dystrophy

**DOI:** 10.1093/braincomms/fcab075

**Published:** 2021-04-11

**Authors:** Rabah Ben Yaou, Pomi Yun, Ivana Dabaj, Gina Norato, Sandra Donkervoort, Hui Xiong, Andrés Nascimento, Lorenzo Maggi, Anna Sarkozy, Soledad Monges, Marta Bertoli, Hirofumi Komaki, Michèle Mayer, Eugenio Mercuri, Edmar Zanoteli, Claudia Castiglioni, Chiara Marini-Bettolo, Adele D’Amico, Nicolas Deconinck, Isabelle Desguerre, Ricardo Erazo-Torricelli, Juliana Gurgel-Giannetti, Akihiko Ishiyama, Karin S Kleinsteuber, Emmanuelle Lagrue, Vincent Laugel, Sandra Mercier, Sonia Messina, Luisa Politano, Monique M Ryan, Pascal Sabouraud, Ulrike Schara, Gabriele Siciliano, Liliana Vercelli, Thomas Voit, Grace Yoon, Rachel Alvarez, Francesco Muntoni, Tyler M Pierson, David Gómez-Andrés, A Reghan Foley, Susana Quijano-Roy, Carsten G Bönnemann, Gisèle Bonne

**Affiliations:** 1 Sorbonne Université, Inserm, Institut de Myologie, Centre de Recherche en Myologie, F-75013 Paris, France; 2 APHP-Sorbonne Université, Neuromuscular Disorders Reference Center of Nord-Est-Île de France, FILNEMUS, ERN-Euro-NMD, Service de Neuromyologie, Institute de Myologie, G.H. Pitié-Salpêtrière Paris F-75013, France; 3 Neuromuscular and Neurogenetic Disorders of Childhood Section, National Institute of Neurological Disorders and Stroke, National Institutes of Health, Bethesda, MD, USA; 4 APHP-Université Paris-Saclay, Neuromuscular Disorders Reference Center of Nord-Est-Île de France, FILNEMUS, ERN-Euro-NMD, Pediatric Neurology and ICU Department, DMU Santé Enfant Adolescent (SEA), Raymond Poincaré University Hospital, Garches France; 5 INSERM U 1245, ED497, School of Medicine, Rouen University, Rouen, France; 6 Department of Pediatrics, Peking University First Hospital, Beijing, China; 7 Neuromuscular Unit, Neuropaediatrics Department, Hospital Sant Joan de Déu, Institut de Recerca Sant Joan de Déu, CIBERER - ISC III, Barcelona, Spain; 8 Neuroimmunology and Neuromuscular Diseases Unit, Fondazione IRCCS Instituto Neurologico Carlo Besta, Milano, Italy; 9 Dubowitz Neuromuscular Centre, UCL Great Ormond Street Institute of Child Health, Great Ormond Street Hospital Trust, London, UK; 10 Servicio de Neurología, Hospital de Pediatría J.P. Garrahan, Buenos Aires, Argentina; 11 Northern Genetics Service, The Newcastle upon Tyne NHS Foundation Trust, Newcastle upon Tyne, UK; 12 Department of Child Neurology, National Center Hospital, National Center of Neurology and Psychiatry (NCNP), Tokyo, Japan; 13 APHP-Sorbonne Université, Neuromuscular Disorders Reference Center of Nord-Est-Île de France, FILNEMUS, ERN-Euro-NMD, Department of Neuropediatrics, Hôpital Armand Trousseau, Paris, France; 14 Paediatric Neurology, Policlinico Gemelli, Fondazione Policlinico Universitario Agostino Gemelli IRCCS, Rome, Italy; 15 Department of Neurology, Faculdade de Medicina da Universidade de São Paulo (FMUSP), São Paulo, Brazil; 16 Pediatric Neurology Department, Clínica Las Condes, Santiago, Chile; 17 John Walton Muscular Dystrophy Research Centre, Institute of Integrated Laboratory Medicine, Newcastle University and Newcastle Hospitals NHS Foundation Trust, Newcastle Upon Tyne, UK; 18 Unit of Muscular and Neurodegenerative diseases, Department of Neurological and Psychiatric science,s Bambino Gesù Children's Hospital, Rome, Italy; 19 Paediatric Neurology Department and neuromuscular Center, Hôpital Universitaire des Enfants Reine Fabiola, Université Libre de Bruxelles, Brussels, Belgium; 20 APHP-Centre - Université de Paris, Neuromuscular Disorders Reference Center of Nord-Est-Île de France, FILNEMUS, ERN-Euro-NMD, Necker-Enfants Malades Hospital, Paris, France; 21 Neurología Pediátrica, Unidad Neuromuscular, Hospital Luis Calvo Mackenna, Clínica Alemana de Santiago, Santiago, Chile; 22 Department of Pediatrics, Pediatric Neurology Service, Medical School, Universidade Federal de Minas Gerais, Belo Horizonte, MG, Brazil; 23 Neurología Pediátrica Hospital Roberto del Río- Universidad de Chile - Clínica Las Condes Santiago, Chile; 24 CHRU de Tours, Université François Rabelais de Tours, INSERM U1253, Tours, France; 25 Department of neuropediatrics, CHU Strasbourg- Hautepierre, Strasbourg, France; 26 Service de Génétique médicale, INSERM, CNRS, UNIV Nantes, CHU Nantes, l'institut du Thorax, Nantes, France; 27 Unit of Neurology, Department of Clinical and Experimental Medicine, University of Messina, Messina, Italy; 28 Cardiomiology and Medical Genetics, Department of Experimental Medicine, University of Campania, Naples, Italy; 29 Children's Neurosciences Centre, Royal Children's Hospital, Victoria, Australia; 30 Service de Pédiatrie A, Neurologie pédiatrique, CHU de Reims, American Memorial Hospital, Reims, France; 31 Department of Neuropediatrics, Developmental Neurology and Social Pediatrics, Children's Hospital 1, University of Duisburg-Essen, Essen, Germany; 32 Department of Clinical and Experimental Medicine, University of Pisa, Pisa, Italy; 33 Department of Neuroscience, Center for Neuromuscular Diseases, University of Turin, Turin, Italy; 34 National Institute for Health Research Great Ormond Street Hospital Biomedical Research Centre, University College London Great Ormond Street Institute of Child Health, London, UK; 35 Divisions of Neurology and Clinical and Metabolic Genetics, Department of Paediatrics, The Hospital for Sick Children, University of Toronto, Toronto, Ontario, Canada; 36 Congenital Muscle Disease International Registry (CMDIR), Cure CMD, Lakewood, CA, USA; 37 Departments of Pediatrics and Neurology and the Board of Governors Regenerative Medicine Institute, Cedars-Sinai Medical Center, Los Angeles, CA, USA; 38 Pediatric Neurology (ERN-RND - EURO-NMD), Vall d’Hebron Institut de Recerca (VHIR), Hospital Universitari Vall d’Hebron, Vall d’Hebron Barcelona Hospital Campus, Barcelona, Spain; 39 INSERM U 1179, University of Versailles Saint-Quentin-en-Yvelines (UVSQ), France; 40 APHP-Sorbonne Université, Neuromuscular Disorders Reference Center of Nord-Est-Île de France, FILNEMUS France, ERN-Euro-NMD, Paris, France

**Keywords:** laminopathies, striated muscle, *LMNA*, early onset, muscular dystrophy

## Abstract

Muscular dystrophies due to heterozygous pathogenic variants in *LMNA* gene cover a broad spectrum of clinical presentations and severity with an age of onset ranging from the neonatal period to adulthood. The natural history of these conditions is not well defined, particularly in patients with congenital or early onset who arguably present with the highest disease burden. Thus the definition of natural history endpoints along with clinically revelant outcome measures is essential to establishing both clinical care planning and clinical trial readiness for this patient group. We designed a large international cross-sectional retrospective natural history study of patients with genetically proven muscle laminopathy who presented with symptoms before two years of age intending to identify and characterize an optimal clinical trial cohort with pertinent motor, cardiac and respiratory endpoints. Quantitative statistics were used to evaluate associations between *LMNA* variants and distinct clinical events. The study included 151 patients (median age at symptom onset 0.9** **years, range: 0.0–2.0). Age of onset and age of death were significantly lower in patients who never acquired independent ambulation compared to patients who achieved independent ambulation. Most of the patients acquired independent ambulation (n = 101, 66.9%), and subsequently lost this ability (n = 86; 85%). The age of ambulation acquisition (median: 1.2 years, range: 0.8–4.0) and age of ambulation loss (median: 7 years, range: 1.2–38.0) were significantly associated with the age of the first respiratory interventions and the first cardiac symptoms. Respiratory and gastrointestinal interventions occurred during first decade while cardiac interventions occurred later. Genotype–phenotype analysis showed that the most common mutation, p.Arg249Trp (20%), was significantly associated with a more severe disease course. This retrospective natural history study of early onset *LMNA*-related muscular dystrophy confirms the progressive nature of the disorder, initially involving motor symptoms prior to onset of other symptoms (respiratory, orthopaedic, cardiac and gastrointestinal). The study also identifies subgroups of patients with a range of long-term outcomes. Ambulatory status was an important mean of stratification along with the presence or absence of the p.Arg249Trp mutation. These categorizations will be important for future clinical trial cohorts. Finally, this study furthers our understanding of the progression of early onset *LMNA*-related muscular dystrophy and provides important insights into the anticipatory care needs of *LMNA*-related respiratory and cardiac manifestations.

## Graphical Abstract

**Graphical Abstract:**
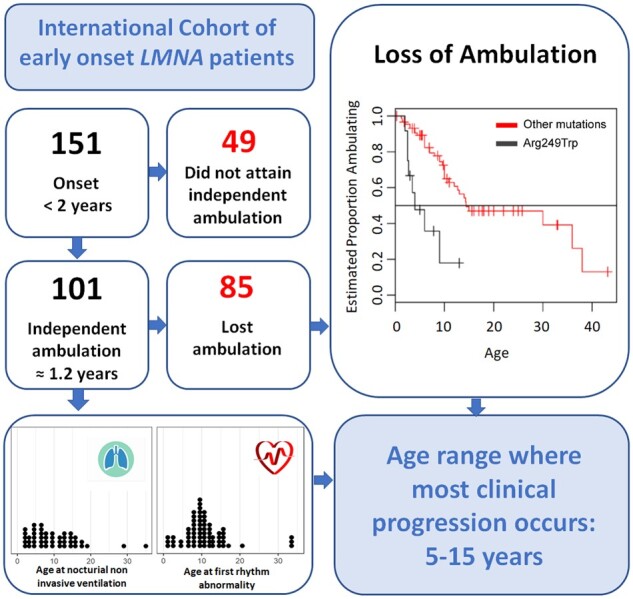

Abbreviated summary

Ben Yaou et al. report the largest cohort of *LMNA*-congenital muscular dystrophy. They identified phenotypic subgroups based on walking status and specific mutation and defined age range where most clinical progression occurs (5–15 years ago). This will help subject stratification and clinical endpoints designing for future clinical trials

## Introduction

Laminopathies are a heterogeneous group of disorders caused by mutations in the *LMNA* gene encoding lamin A/C. These disorders affect either specific tissues (i.e. striated muscle, adipose tissue or peripheral nerve) or present as more systemic disorders such as premature aging syndromes.^1^ Laminopathies involving striated skeletal and cardiac muscles were first recognized 21 years ago, and include Emery–Dreifuss muscular dystrophy (EDMD), limb-girdle muscular dystrophy type 1B (previously known as LGMD1B) and isolated dilated cardiomyopathy with conduction system defects and arrhythmias (DCM-CD).^2–4^ When skeletal muscle is affected, these disorders typically manifest as childhood or young adult onset myopathies with or without joint contractures and rigid spine followed by cardiac disease emerging within the second or third decade of life.^5^^,^^6^

An early infantile-onset of striated muscle laminopathy, so-called *LMNA*-related congenital muscular dystrophy (L-CMD) can also occur. L-CMD represents a relatively recently characterized disease group.^5–9^ While this group is phenotypically diverse, it can be operationally defined as having an onset of skeletal muscle manifestations within the first two years of life, when early motor development that includes walking and running should typically be attained.^9^ It is of great medical importance to distinguish this group of patients from other later onset *LMNA*-related phenotypes (EDMD, LGMD1B), generally beginning after 2 years of age, not only because of their early and typically more severe motor manifestations but, most importantly, because of the potential of early life-threatening complications involving nutritional, respiratory and cardiac compromise. In addition, orthopaedic complications including severe scoliosis may also have severe functional consequences and life-threatening respiratory complications for which surgery may be needed.^10–13^ Even though there is a recognizable clinical phenotype that includes prominent axial weakness, motor delay and/or rapid motor development loss with severe neck weakness (dropped head), there is little understanding of the natural history of this important subgroup of patients. To date, only case reports or large series of patients have been reported in which authors described the skeletal muscle, respiratory, cardiac and gastrointestinal manifestations at the individual level.^6^^,^^7^^,^^9–17^ In a recent report, Fan et al. showed that the proportion of their L-CMD patients who achieved ambulation had a significantly earlier ambulation loss than patients with EDMD or LGMD1B. A detailed chronology of the disease progression and the disease events over time will assist in clinical management by allowing preventive or proactive medical interventions to delay or limit complications as well as help in identifying appropriate outcome measures for distinguishing populations with different outcomes. Targeted therapeutic interventions for the laminopathies as a group are currently advancing to clinical trials. Examples include the use of MAP kinase pathway inhibitors to slow the progression of the cardiac dysfunction (reviewed in Macquart et al.^1^, *Clinicaltrial.gov* No. NCT03439514; *Clinicaltrialsregister.eu* No. 2017-004310-25), trials of nicotinamide adenine dinucleotide^18^ and N-acetyl cysteine.^19^ Given these advances in the development of potential therapeutics, improving clinical trial readiness is of timely importance.

This retrospective natural history study was performed to provide a well-documented dataset from a large international cohort of patients by collecting patient genotypes and associated clinical information documenting disease involvement, progression and complications. The main goals of this study were: (i) to identify phenotypic subgroups for stratification of subjects for future clinical trials and (ii) to define the age range during which the most consistent clinical progression occurs to assist with designing clinical endpoints for effective clinical trials. This large, international natural history study helps to define a clinical trial population and relevant endpoints while allowing for the design of a prospective natural history study or a run-in study for an interventional one.

## Materials and methods

### Participating centres, inclusion criteria, clinical datasheet and patients identification

A cross-sectional retrospective data collection was performed on an international cohort of neuromuscular patients from 34 referral centres (Supplementary Table 1). Phenotypic and genotypic data were collected from patients with a confirmed pathogenic variants in the *LMNA* gene and symptoms involving skeletal muscle with a maximum age of onset of two years (regardless of the patients’ detailed phenotypic classification otherwise) within the period ranging from the birth to December 2015. For each patient, information concerning demographics (sex, date of birth, referring centre, clinician, country of referring neuromuscular clinic, country of origin, date of last visit, date of death if applicable), detailed genetic results, phenotypic characterictics and major clinical interventions were collected (detailed in Supplementary Table 2). Patient information was sent to one of the authors (RBY) and gathered in a single dataset file where each patient was identified by an anonymous number.

### Clinical definitions: Cardiac and orthopaedic abnormalities and interventions

Cardiac abnormalities refer to any significant ECG/Holter-ECG/Insertable Cardiac Monitor (ICM) anomalies (abnormal P wave, permanent or paroxysmal conduction defects, permanent or paroxysmal supraventricular or ventricular arrhythmias) and echocardiography abnormalities (left or right ventricular dysfunction, abnormal systolic or diastolic function, abnormal fractional shortening, abnormal chamber dimensions, pulmonary hypertension, regional dyssyncrony and strain abnormalities). Orthopaedic abnormalities refer to the presence of joint contractures involving any joint and the presence of scoliosis. Major interventions refer to those main medical interventions for disease complications including: gastrointestinal (gastrostomy feeding tube), orthopaedic (non-surgical and surgical treatments for joint contractures and scoliosis), respiratory (intermittent positive pressure breathing or IPPB, non-invasive ventilation or NIV and tracheostomy) and cardiac (medications, pacemaker or implantable cardioverter defibrillator (ICD) insertion). First cardiac abnormalities refer to the first cardiac anomalies documented in the course of the disease (via ECG, Holter-ECG, ICM or echocardiography). First respiratory or cardiac interventions refer to the first major interventions documented in the course of the disease including heart medication, pacemaker or ICD implantation, IPPB, NIV or tracheostomy.

### Statistical analysis

Demographic and basic clinical information were described using median and range. The distribution of common *LMNA* variants was grouped by exon with corresponding nucleotide change and analysed by ages of intervention, symptom onset and loss of ambulation using ANOVA. Age of onset was described by maximal motor function groups and analysed using ANOVA, as well as across different mutation types. Age at first interventions and abnormalities were described using median and range and explored for cross-correlations using Pearson’s or Spearman’s correlations. Numbers of patients with orthopaedic abnormalities are described as numbers (percent). Kaplan–Meier curves were used to describe the time-to-loss of ambulation and other time-to-event variables, using the age at time of event if the event had occurred, or the patient’s age at last visit if the event had not yet occurred. We also used Kaplan–Meier curves to explore differences in time-to-event data by ambulation status and by presence of the *LMNA* p.Arg249Trp variant versus other variants. Steroid usage was described and investigated graphically. Missing data were not imputed and all available data were used where possible. Statistical analyses were conducted using R version 3.5.0.

### Data availability

The authors confirm that the data supporting the findings of this study are available within the article and its Supplementary Material. A detailed Excel spreadsheet of the clinical data is available upon request.

## Results

The original patient cohort consisted of 190 patients (112 males; 78 females) originating from 23 different countries and 34 participating referral centres located in 14 different countries (geographic representation in Supplementary Fig. 1). All patients had symptom onset at less than 5 years regardless of mutation state, i.e. hetero-, homo- or compound heterozygous (Supplementary Table 1). The dataset was further screened for children who were younger than 2 years of age at the time of their first clinical manifestations associated with skeletal muscle dysfunction - thus consistent with the clinical definition of L-CMD - and who possessed a confirmed causative heterozygous variant in the *LMNA* gene (Supplementary Table 1). This resulted in a final cohort of 151 patients, most of whom resided in France, UK and China (17.8%, 15.9% and 14.6%, respectively). Hereafter, the study cohort refers to these 151 patients.

### Overall patient characteristics and disease progression

Summary of clinical characteristics of the 151 patients (94 females/57 males) are shown in Table 1 according to their maximal motor achievements when relevant. Eighty-one of the patients had been diagnosed as L-CMD (60 showing the classical dropped head syndrome), 66 as EDMD and the remaining four as LGMD1B. They were followed during a median period of 8 years (range: 0.1–41.2). The median age at the time of the last visit was 9 years (range: 0.1–43.2) and the median age of symptom onset (first recognition of disease manifestations) was 0.9 years (range: 0.0–2.0). Age of onset of patients who achieved independent ambulation is significantly higher than patients who never achieved independent ambulation (medians were 1 year (range: 0–2) and 0.1 year (range: 0–1.5), respectively, *P* < 0.001). Most patients achieved independent ambulation (n = 101; 67%) at a median age of 1.2 years (range: 0.8–4.0). In those patients in whom a precise age at ambulation loss was available (n = 43), the reported median age was 7 years (range: 1.2–38.0). Among the 49 patients who never achieved independent ambulation, 36 walked with support or sat, five crawled and eight never acquired any classical motor milestones (data not shown). The 20 patients in this cohort who had passed away died at a median age of 12.1 years (range: 0.4–40.0) with a significant difference between those who achieved independent ambulation (10 patients) and those who did not (10 patients) with a median age of death of 17.5 and 7.9 years, respectively (*P* = 0.002). Six patients had a pacemaker, and four patients had ICD at the time of death. Unfortunately, detailed causes of death were not available to distinguish those patients who died from cardiac or extracardiac causes.

**Table 1:** Patient’s overall characteristics and major disease progression markers, interventions and cardiac first significant abnormalities according to their ambulation status

		Median age in years (range in years)/*N* or *N* (%)^a^						
		All patients (*N* = 151 patients^b^)		Patients who acquired independent walk (101 patients)		Patients who never acquired independent walk (49 patients)		*P* value
Overall motor course and survival characteristics								
Age at last visit	9.0 (0.1–43.2)/151^b^		11 (0.3–43.2)/101		5 (0.1–20.0)/49		<0.001	
Age of onset	0.9 (0.0–2.0)/151^b^		1.0 (0–2)/101		0.1 (0–1.5)/49		<0.001	
Age at independent ambulation	1.2 (0.8–4.0)/101		1.2 (0.8–4.0)/101		–		–	
Age at loss of ambulation *(for those who achieved independent ambulation)*	7 (1.2–38.0)/43		7.0 (1.2–38.0)/43		–		–	
Age at loss of sitting *(for those who never achieved independent ambulation)*	2.5 (0.83–3.0)/6		–		2.5 (0.83–3.0)/6		–	
Age at death	12.1 (0.4–40.0)/20		17.5 (9.6–40.0)/10		7.9 (0.4–14.4)/10		0.002	
Age at ICD implantation	17.0 (8.0–40.0)/12		17.0 (8.0–40.0)/12		0		–	
Age at pacemaker implantation	17.0 (2.0–32.6)/11		18.0 (14.0–32.6)/9		6.5 (2–11)/2		0.04	
Gastrointestinal and respiratory interventions								
Age at gastrostomy feeding tube	5.0 (1.8–16.5)/15		8.5 (1.8–16.5)/8		3.5 (2–7.9)/7		0.09	
Age at first respiratory intervention	7.0 (1.0–35.0)/62		9.0 (3.0–35.0)/40		3,8 (1.0–18)/22		<0.001	
Age at non-invasive ventilation	8.5 (1.6–40.0)/48		12.5 (5.0–40.0)/29		4 (1.6–18)/19		<0.001	
Age at tracheostomy	6.0 (0.0–35.0)/13		11.0 (4.5–35.0)/6		4.5 (0.0–23.0)/7		0.12	
Orthopaedic abnormalities (scoliosis, joint contractures) and interventions								
Presence of scoliosis^c^	77 (51.0)		54 (53.5)		23 (46.9)		0.69	
Age at scoliosis non-surgical treatment	4.5 (1.0–15.0)/20		5.0 (2.0–15.0)/13		3.0 (1.0–7.8)/7		0.09	
Age at scoliosis surgical treatment	13.0 (8.0–17.0)/14		13.1 (8.0–17.0)/12		12.7 (12.3–13.0)/2		0.22	
Presence of only one joint contracture^c^	16 (10.6)		11 (10.9)		5 (10.2)		1.00	
Presence of more than one joint contracture^c^	112 (74.2)		78 (77.2)		34 (69.4)		0.32	
Age of first surgical joint contractures therapy	7.0 (0.1–16.0)/34		7.0 (3.0–16.0)/29		6.0 (0.1–8.0)/5		0.37	
Cardiac abnormalities (ECG, Holter-ECG, ICM and echocardiography) and interventions								
Age at first cardiac abnormality		9.3 (0.2–34.0)/73^b^		10 (0.2–34.0)/55		8 (1.3–14)/17	0.02	
Age at first rhythm abnormality		10.0 (0.6–34.0)/66^b^		11 (3–34.0)/49		8.5 (1.3–14)/16	0.01	
Age at first echocardiography abnormality		11.0 (0.2–36.0)/33^b^		12.0 (0.2–36.0)/26		9.3 (3–14)/6	0.31	
Age at first flattening of P wave		10.4 (3.0–34.0)/22		12 (3.0–34.0)/17		10.0 (3–18.8)/5	0.91	
Age at first PR interval prolongation		14.0 (5.0–33.0)/25		14 (5.0–33.0)/21		11.6 (10–13)/4	0.30	
Age at first cardiac intervention		12.0 (2.0–40.0)/42		13.0 (6.0–40.0)/31		9.6 (2–18.8)/11	0.01	
Age at first heart medication		11.0 (2.0–35.0)/34		13.0 (6.0–35.0)/24		9.3 (2–18.8)/10	0.03	

For the definition of ‘cardiac abnormalities’, ‘interventions’ and ‘first’ cardiac or respiratory interventions, see ‘Materials and methods’ section. *P* value refers to Wilcoxon rank sum for continuous variables or Fisher’s exact test for categorical variables for the test between ambulant and never ambulant groups.

ECG, electrocardiography; ICM, insertable cardiac monitor (LINQ or others).

a *N* refers to the number of observations that were available at the time of analysis.

b For one patient, the corresponding clinical characteristics (maximal motor achievements or cardiac abnormalities) were unknown explaining why total patient numbers are not equal to the sum for the two subgroups.

c Note that these numbers are expressed in number of observations (percent of either total cohort or total patients in each subgroup).

**Figure 1:**
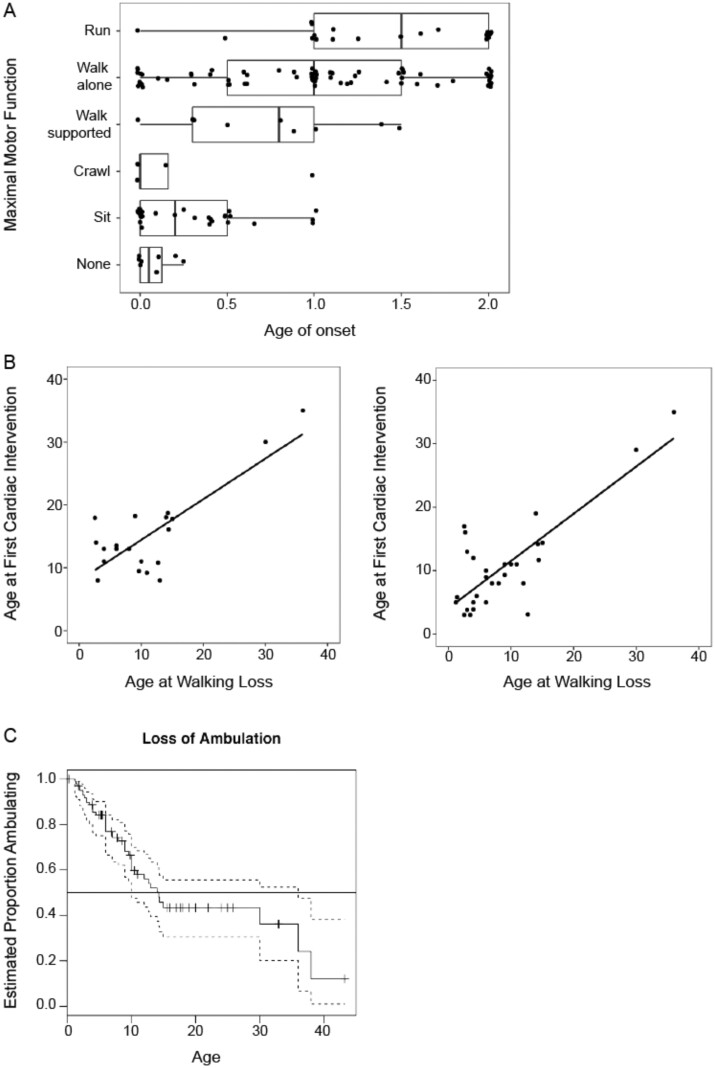
**Clinical characteristics of the cohort.** (**A**) Maximal motor function achieved for individuals at a given age of onset. This demonstrates that individuals with an earlier age of onset may still reach a maximum functional ability of independent ambulation; however, those with later ages of onset tend to have better maximal motor function (*P* < 0.01). (B) Age at first cardiac intervention (left panel) and age at first respiratory intervention (right panel) mapped against age at loss of ambulation. Although there is a strong relationship when all data is included (*r* = 0.80, *P* < 0.01; *r* = 0.81, *P* < 0.01, respectively), this relationship is greatly reduced after removal of the two individuals with late interventions (*r* = 0.22, *P* = 0.37; *r* = 0.38, *P* = 0.04, respectively). (C) Kaplan–Meier time to event analysis for loss of ambulation. About half of the individuals who acquired independent ambulation lost this ability by age 14 years.

We started by a basic global analyse. We found no strong differences in key clinical variables between males and females. Maximal motor function was significantly related to the age of onset (ANOVA; *F*(5, 143)=16.3; *P* < 0.01) with the ability to run appearing more highly associated with a later onset of disease (after one year) (Fig. 1A). Analysis of clinical features and interventions (orthopaedic, respiratory, cardiac and gastrointestinal) by age of occurrence, showed that orthopaedic abnormalities (scoliosis and joint contractures), orthopaedic non-surgical interventions, surgical treatments of joint contractures, respiratory interventions (NIV and tracheostomy) and gastrointestinal interventions (gastrostomy feeding tube) generally occurred during the first decade of life, while the first cardiac abnormalities and interventions were detected at the transition from the first to second decade of life (Table 1). Scoliosis surgical treatment occurred at a median age of 13 years (range: 8–17).

### Spectrum of *LMNA* mutations, creatine kinase levels and muscle biopsy results

**Figure 2:**
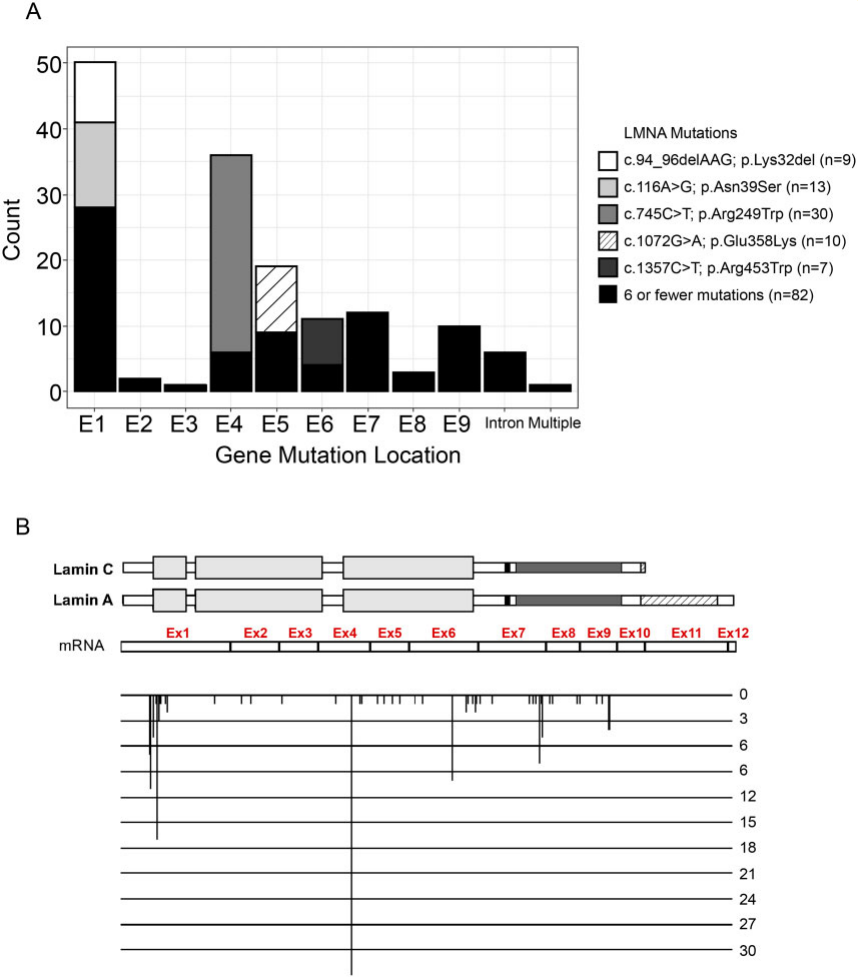
**Map of heterozygous *LMNA* variants identified in the 151 patients with onset before 2 years of age.** (**A**) Distribution of variants in the different exons of the *LMNA* gene. The *LMNA* c.745C>T, p.Arg249Trp variant was found in 30 patients, the c.116A>G, p.Asn39Ser in 13, the c.1072G>A, p.Glu358Lys in 10, the c.94_96delAAG, p.Lys32del in 9 and the c.1357C>T, p.Arg453Trp in 7. Variants identified in six or fewer patients are depicted together. (**B**) Mutation map at the protein structure level. Mutations are depicted and aligned according to their position on both exons and lamins A and C protein domains.^20^ The height of each mutation position corresponds to the number of patients carrying mutation at the specific position.

Sixty-six different *LMNA* variants were identified (52 missense, 5 splice site, 6 small in-frame intra-exonic deletions, 1 small duplication of 3 nucleotides and 2 small intronic deletions (Supplementary Table 3). For these latter two, one corresponds to a 10 nucleotide deletion overlapping exon 8-intron 8 that abolishes the splice donor site of intron 8 and results in an in-frame deletion of the last codon of exon 8 by creating a new splice site 4 nucleotides upstream the end of exon 8; and the other is a 8 nucleotide deletion in intron 8 near the intron 8-exon 9 junction leading to exon 9 skipping. The distribution of common *LMNA* variants by exon with corresponding nucleotide and amino acid changes at the protein level is shown in Fig. 2. At least seven individuals were present in each of the groups that correspond to five common mutations (p.Lys32del, p.Asn39Ser, p.Arg249Trp, p.Glu358Lys, p.Arg453Trp) with the p.Arg249Trp emerging as the single most common *LMNA* variant in this series (30 patients, 20%). Regarding the transmission of identified mutations, 54 occurred *de novo*, 14 were inherited from one of the parents. In the remaining 83 patients where the parents were not analysed, in 76 no familial history of skeletal or cardiac muscles disorders was found, thus suggesting a possible *de novo* occurrence, while family history was not available in six patients. In this study cohort, we did not analyse variants that were present in fewer than seven individuals for phenotype/genotype analysis due to their small sample size.

The median creatine kinase value among the patients in whom creatine kinase value was available was 1132 (range from normal levels to 2700 IU/l) in the 45 patients who did not achieve independent ambulation and 900 (normal level to 3107 IU/l) in the 82 patients who could ambulate independently. Muscle biopsy was performed in 84% of patients (127/151) at a median age of 3 years (range: 0–35 years) and displayed either dystrophic findings (76%; 96/127), non-specific myopathic findings (22%; 28/127), inconclusive findings in two patients and normal findings in one. Signs of inflammation were seen in 31% of all biopsies (39/127). Eighteen (50%) of these inflammatory muscle biopsies were found in patients carrying three different *LMNA* variants (p.Arg249Trp, *n* = 8; p.Glu358Lys, *n* = 5 and p.Asn39Ser, *n* = 5). Unfortunately, no enough data were available in the dataset to specify what kind of inflammatory cells, their localization or HLAI-I staining signs were found.

### Correlations in non-ambulating patients

Non-ambulating patients showed an earlier disease progression affecting gastric, respiratory and cardiac complications that began in infancy (Table 1) associated with further loss of residual motor function. In fact, among the 36 patients whose maximal motor milestone was sitting or walking with support, six lost the ability to sit at a median age of 2.5 years (range: 0.83–3) (Table 1). Orthopaedic abnormalities in non-ambulant patients did not occur significantly more frequently than in ambulatant patients. While there is a trend towards statistical significance for non-surgical treatments of scoliosis (*P* = 0.09) (Table 1), surgical and non-surgical treatments of scoliosis and joint contractures were not significantly more frequent in non-ambulant patients.

**Figure 3:**
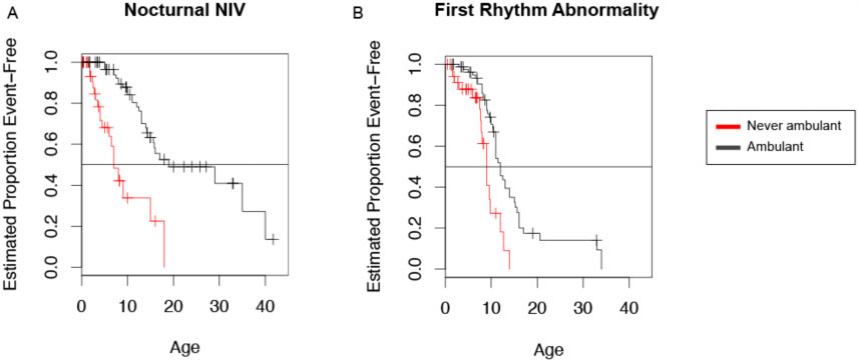
**Time-to-event analysis.** (**A**) Time-to-event analysis to NIV (non-invasive ventilation) and (**B**) first rhythm abnormality demonstrate that these events are reached earlier in patients who were never ambulatory when compared to those who had achieved ambulation.

Comparison of time-to-event between patients who ambulated and those who never ambulated independently showed that the latter had an earlier onset of cardiac rhythm abnormalities (Kaplan–Meier median ages 9 and 12, respectively, *P* < 0.001) (Fig. 3A) and an earlier initiation of NIV (Kaplan–Meier median ages 7 and 19, respectively, *P* < 0.001) (Fig. 3B) in the years following their first decade. Thus, the inability to achieve independent ambulation is associated with a worse respiratory and cardiac prognosis.

### Correlations in ambulant patients

The age at first cardiac and first respiratory interventions initially show a linear correlation across all subjects, relating to age at loss of ambulation (0.81 cardiac (95% confidence interval (CI) 0.57–0.92); *P* < 0.001; 0.80 respiratory (95% CI 0.62–0.90); *P* < 0.001). However, these relationships were strongly determined by two older individuals with later interventions (Fig. 1B). When removing these two patients, the correlation in the remaining cohort is less strong (Pearson correlations: 0.22 cardiac (95% CI −0.27 to 0.63); *P* = 0.37; 0.38 respiratory (95% CI 0.01–0.67); *P* = 0.01).

**Figure 4:**
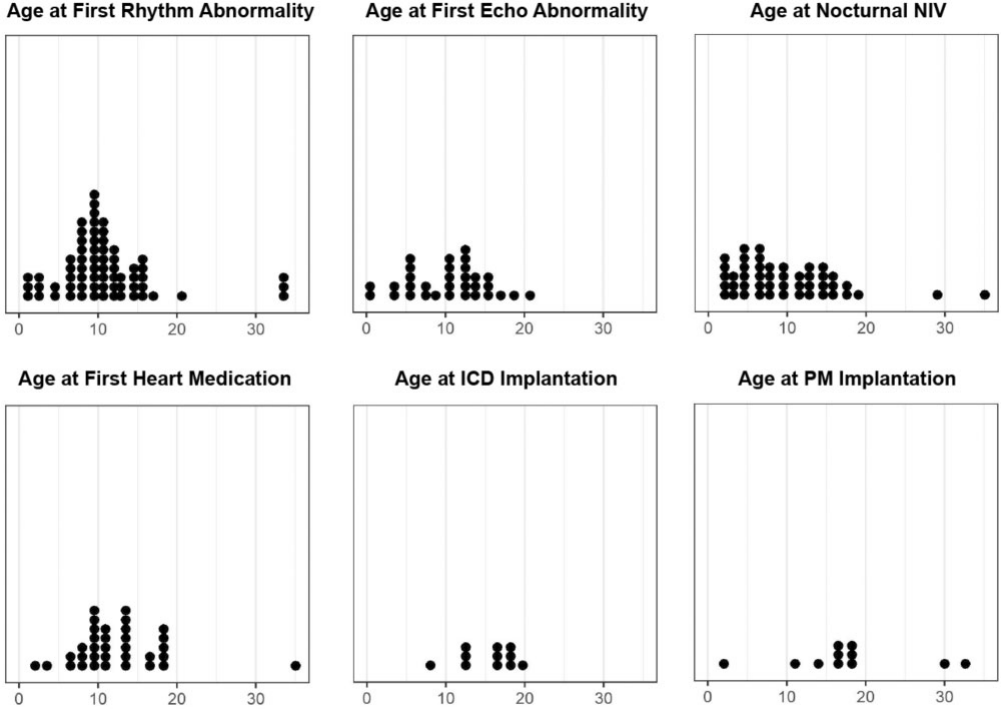
Histogram plot of ages for disease-related symptoms and interventions. An initial rhythm abnormality (46% of the cohort) was seen at a median estimated age of 11 years old, followed by the initiation of NIV (non-invasive ventilation) at a median estimated age of 16 years. Cardiac medication was taken by 25% of the cohort with echocardiogram abnormalities present in 22%. ICD and pacemakers were relatively uncommon (9% and 7%, respectively).

Time-to-event analysis was performed to highlight the age range at which defined clinical events were most likely to occur. For example, of the 101 individuals who had acquired independent ambulation mostly in the normal age range, 86 subsequently lost the ability to walk, 43 had an available age at loss of ambulation. An estimated half of these patients had lost ambulation by 14 years of age (Fig. 1C). An apparent plateau in the time to loss of ambulation analysis occurred due to age censoring and limited observations at older ages (Fig. 1C). Histogram plots were used as a visual aid and helped in identifying age 5–15 years as the age range during which most clinical events and interventions occurred (Fig. 4), and thus the range of the most noticeable clinical progression in ambulant patients.

### Genotype–phenotype correlation

**Figure 5:**
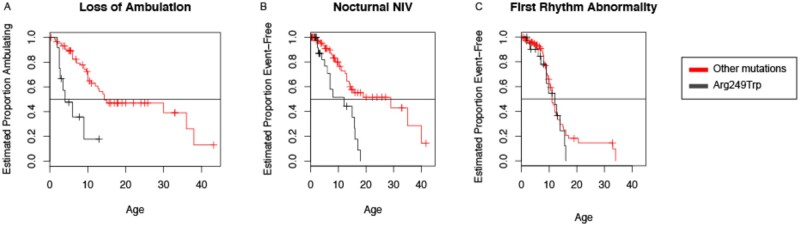
**Genotype–phenotype analysis stratified time-to-event analysis for the most common variant (p.Arg249Trp) against all other variants.** (**A**) Visual analysis of the curve for loss of ambulation suggests that half of individuals lose ambulation by five years of age for patients with the p.Arg249Trp variant. (**B**) A similar difference is shown for time to NIV (non-invasive ventilation) (*n* = 12 p.Arg249Trp, *n* = 29 other variants). (**C**) No difference is noted for time to first rhythm abnormality (*n* = 12 p.Arg249Trp, *n* = 11 other variants).

A detailed genotype–phenotype analysis was limited given that most of the individual *LMNA* variants, of the 66 different *LMNA* variants present in our cohort, were reported in a small number of patients. No statistical significance was reached when comparing specific mutations to the age of symptom onset. Furthermore, there was no statistical significance detected between specific mutations and either the age at first intervention or the age at loss of ambulation. The most frequent variant, p.Arg249Trp, affected 30 patients and was of particular interest. Nineteen patients (63%) with the p.Arg249Trp variant never acquired independent ambulation, while 11 (37%) did. When stratifying the time-to-event analysis for the p.Arg249Trp variant against all other variants, Kaplan–Meier curves for loss of ambulation suggests that 50% of individuals with the p.Arg249Trp variant lost ambulation by five years of age versus a loss of ambulation 10 years later for patients with other *LMNA* variants (Fig. 5A). There is a similar difference present with the time to NIV initiation (median age = 12 years for patients with the p.Arg249Trp variant; median age = 29 years for patients with other *LMNA* variants). No such difference was observed for time to first rhythm abnormality (median age = 12 years for patients with the p.Arg249Trp variant; median age = 11 years for patients with other *LMNA* variants) (Fig. 5B and C). Overall, these results suggest that the *LMNA* genotype p.Arg249Trp is associated with a worse clinical prognosis.

### Outliers

There were five patients with symptom onset within two years of life who were noted as outliers (2 SD above the mean; also confirmed visually) with regards to at least one key clinical variable (Supplementary Table 4). The outlier status of these five patients was a result of overall intervention variables, age at first cardiac abnormality and age at loss of ambulation.

### Steroid use

There were 22 patients who were treated with corticosteroids, with 16 patients treated for six months duration or longer. Fifty percent (11/22) of these patients were still ambulant at their last study visit (median age = 9.75 years; range: 1.0–33.0). No significant patterns or deviations from the rest of the cohort were present in graphical analysis of subjects who received steroids. Due to the limited longitudinal data availability for these patients (timed tests and respiratory functional tests) before and after corticosteroids use, we were not able to further investigate the potential effect of corticosteroids on motor and respiratory function.

## Discussion

This international retrospective natural history study consists of a cohort of 151 L-CMD patients as defined by the onset symptom before two years of age and currently represents the largest clinical dataset of patients with early onset laminopathies specifically affecting the striated muscle. This cohort represents a subgroup of laminopathy patients of particular medical needs given the severe clinical manifestations and the relatively rapid progression of symptoms. Since the early 2000s, several reports of laminopathies had included cases of early-onset laminopathy.^6–8^^,^^12^^,^^14^^,^^21^ Benedetti et al.^7^ highlighted that a subgroup of patients with childhood onset (2.4±2.3 years old) had predominant scapuloperoneal and facial weakness. These patients typically had non-truncating variants (missense, small in-frame deletions) in *LMNA*, producing a full-length lamin A/C protein with a dominant-negative mechanism. The most severely affected patients of this spectrum of the striated muscle laminopathies were classified as L-CMD by Quijano-Roy et al.,^9^ a severe congenital muscular dystrophy characterized by infantile-onset of disease (<1 year of age) with absent or delayed ambulation, early loss of ambulation, early respiratory insufficiency requiring NIV or ventilation via a tracheostomy. This clinical phenotype of L-CMD has been validated in several subsequent reports that sometimes also been reported to include early and severe cardiac involvement indicating that this group of patients is in urgent need of development of therapeutic interventions.^10^^,^^11^^,^^13^^,^^15–17^^,^^22–35^ The recent advances in the development of potential therapeutics for striated muscle laminopathies in general and for L-CMD in particular have highlighted the need for a more in-depth characterization of the natural history of L-CMD in order to identify phenotypic subgroups for stratification of subjects, to define the age range during which the most consistent clinical progression occurs and to detect suitable outcome measures and endpoints that can be used in future clinical trials.

All participants fulfilled the defining inclusion criterion of manifestation of striated muscle involvement, disease onset before two years of age, and the presence of a confirmed pathogenic heterozygous variant in *LMNA.* Data were collected blindly without bias towards the severity or subsequent course of the disease or any other prior phenotypic classification. We constructed a natural history chronology by using time-to-event analyses and histogram plotting that allows for speculation regarding the age range during which most of the disease progression and the definition of medical endpoints can be predicted in the majority of patients. The goal was to define phases of clinical progression measured by loss of major functional motor abilities and the occurrence of medically important events/endpoints such as the initiation of ventilatory support and cardiac events. These events assume importance as study endpoints because of their relevance to morbidity and mortality in this cohort. These data thus have the potential to predefine a potential clinical trial target population in which additional prospective natural history and outcome measure validation would be of the greatest benefit for clinical trial planning and power calculations.

There were several significant clinical associations present in these patients. Firstly, age of onset and age of death were both significantly lower in patients who never aquired independent ambulation. Another significant association is the significantly earlier onset of cardiac rhythm abnormalities and earlier initiation of NIV in patients who did not achieve independent ambulation. This is an interesting result because previous descriptions of congenital laminopathies had suggested two subgroups of patients depending on their clinical presentation.^9^ In this current study, as in prior reports, the two groups of severity are still identified; however, the groups are not defined by age of onset. This may be explained because of the retrospective nature of this study and challenge of precisely recording the timing of the first symptoms. In fact, in this study, no differences were observed in subsequent phenotype depending on the age at symptom onset.

Those patients who never walked independently showed an earlier disease progression that began in infancy and had a more rapid course of loss of residual motor function and the development of life-threatening respiratory and cardiac events in the years following the first decade. Alternatively, those who achieved independent ambulation typically achieved this milestone within a timeframe of normal motor development and typically had a less severe initial disease progression. This ambulatory subgroup typically lost ambulation by late adolescence, subsequently developing cardiac issues or the initiation of chronic ventilatory support. Notably, patients who achieved independent ambulation, but lost ambulation at an earlier age also required earlier respiratory (*P* < 0.001) and cardiac (*P* < 0.001) interventions.

Early cardiac interventions were usually associated with earlier respiratory interventions (*P* < 0.001), with respiratory interventions usually occurring before the cardiac interventions. Thus, clinical severity was positively correlated across the triad of skeletal muscles, respiratory muscles and cardiac muscle (myocardium) without significant disconnects, pointing towards a possible inter-dependence between these three functional systems in this subgroup of laminopathy patients. Correlation to cardiac device placement (due to cardiac conduction abnormalities or malignant arrhythmias) is less strongly linked with the timing of respiratory interventions and the progression of skeletal muscle weakness, which may be due to the small number of patients in which an ICD device had been placed and may also be due to the fact that ICD devices are less used in young children due to anatomic issues and device size incompatibility. Unfortunately, circumstances and causes of death were not available for further analyses. In general, it is evident from this dataset that respiratory interventions occur at a younger average age than cardiac interventions and that loss of ambulation typically occurs before either respiratory or cardiac interventions. This finding confirms and statistically solidifies observation in different reports cited earlier.

For major orthopaedic complications (joint contractures and scoliosis), apart from emerging early in the course of the disease (during the first decade), there are no significant differences between ambulant and non-ambulant patients in terms of the presence of joint contractures and scoliosis, the progression of joint contractures and surgical and non-surgical treatments. This point deserves to be further investigated in future studies with a specific survey especially for scoliosis progression as capturing trustable data in a retrospective study is challenging.

Arguably, the most important analysis in our study for help to define a future clinical trial cohort is the time-to-event and histogram analysis. Typically, time-to-event analysis is used with longitudinal, prospectively acquired data; however, we mimicked this effect by using the binary clinical report of whether or not the event had occurred with retrospectively reported ages. Future studies analysing the plausibility of clinical trials using milestones as outcomes and the relationship of milestones with longitudinal evolution of motor scales and respiratory and cardiac biomarkers are therefore needed for clinical trial readiness in L-CMD.

Several aspects remain to be clarified for this group of patients and are all avenues for future studies. The circumstances of death in these patients should be investigated further by reviewing the clinical contexts of deaths and the data issued from Holter-ECG and ICM tools which were not available for the majority of non-surviving patients of this cohort. The effect of corticosteroids deserves to be studied under a strict therapeutic protocol with predefined longitudinal motor timed tests, and other imaging, respiratory and cardiac systematic investigations to detect any potential favourable effect of this type of treatments. In this context, the inflammatory abnormalities observed in certain patients should be better dissected in order to better characterize the inflammatory process and to put it in parallel with a possible response or non-response to corticosteroid treatment. Finally, special attention should be paid to outlier patients. Their particular phenotype could be the reflection of modifying genes or other modulatory elements of the phenotype which it would be interesting to explore.

From the pathophysiologic point of view, and as highlighted by Benedetti et al..,^7^ the pathogenic variants identified in this cohort are essentially non-truncating variants (missense variants, small in-frame intra-exonic deletions or insertions, small intronic deletions or point mutations affecting splice sites leading to inframe exon deletions) which are expected to produce the synthesis of full-length or quasi-full-length mutant lamin A/C proteins that likely act through a dominant-negative mechanism. We recently reported in the L-CMD mouse model KI-*Lmna*^delK32^ that a similar *Lmna* variant, i.e. small in-frame deletion of one amino acid acted through both decreased expression and mislocalization of the mutated Lamin A/C.^36^ One can hypothesize that a combination of various degrees of haploinsufficiency and dominant-negative effects may contribute to the severity of the disease, as truncating *LMNA* mutations (nonsense and frameshift variants) leading to haploinsufficiency are more commonly associated with isolated cardiac diseases or the less severe forms of the striated muscle laminopathies.^7^^,^^37^^,^^38^ The most common variant our cohort, an Arg249Trp substitution, was primarily associated with congenital and early onset laminopathies and has not been associated with later-onset clinical subttypes (EDMD or LGMD1B) (www.umd.be/LMNA/, *GB and RBY personal communication*). This finding is similar to the one reported by Fan et al.^25^ in a large series of Chinese patients with 12/41 of their patients with L-CMD patients carrying this substitution. Other in-frame variants in our cohort (p.Asn39Ser, p.Lys32del, Glu358Lys, p.Arg453Trp) were less exclusively associated with laminopathies showing congenital or early-onset presentations (only 83.3%, 70%, 63.3% and 0% of published carriers showing congenital/early onset laminopathy, respectively). The Arg249 residue is located within the coil2 domain of Lamin A/C (aa 243–383) at *a* position of a heptade, which is important for hydrophobic interactions between two lamin A/C molecules during filament assembly.^36^^,^^39^^,^^40^ To date, the Arg249 has been reported to be substituted with either a tryptophane (*n* = 39, all with L-CMD with 30 being part of the present study cohort) or glutamine (*n* = 34, 28 EDMD, 5 LGMD1B and 1 L-CMD) (www.umd.be/LMNA/, *GB and RBY personal communication*). One can speculate that modifying the charged arginine residue with the aromatic chain of tryptophan would perturb lamin A/C-lamin A/C interaction in the assembly process more severely, compared to an uncharged polar glutamine residue. Our data indicate that p.Arg249Trp variant to be a major determinant of a severe form of congenital laminopathy (never achieving independent ambulation or experiencing an early loss of ambulation and associated with earlier respiratory interventions) and will be an important consideration for inclusion when designing clinical trial cohorts.

This study had some limitations. Patients recruitment was performed mainly through different paediatric neurology departments that, depending on the centre, may only recruit severe patients with the earliest neuromuscular, respiratory, gastrointestinal and cardiac symptoms and interventions, which may have resulted in biasing the cohort towards severe cases. The age of onset of symptoms was reported by the patients’ parents, which may result in inaccurate estimates, since the recognition of symptoms may not coincide with the onset of symptoms. The use of corticosteroids at the time of interventions or treatment in some patients (e.g. NIV, ICD implantation and cardiac medication) may further skew our results; however, the potential effects of corticosteroids may not be consistent and thus require additional data from prospective controlled studies. The retrospective nature of data collection with its two years age limit for symptom onset may lead to the introduction of phenomena that are likely an artefact of this type of data collection. For instance, the apparent clustering of onset around one and two years of age likely is a product of the self-reporting in which dates are usually estimated by caregivers. Finally, similar caution should be exercised in the time-to-event analyses due to variability in medical practices at the various medical centres that may result in variability of the timing of an intervention. Finally, with age, there are also less-surviving patients which may introduce a possible ‘censoring’ bias in the time-to-event analyses.

Although this study is retrospective and lacks systematic longitudinal data in individual patients, the strength of this dataset is the size of the cohort and the amount of data collected from patients with L-CMD of varying ages. These natural history details reported in this cohort had been largely unknown previously.

## Conclusion

This retrospective analysis of a large international cohort of patients with early onset striated muscle laminopathies expands our understanding of the progression of early infantile-onset striated muscle laminopathy and helps in the anticipatory care of respiratory and cardiac manifestations. Even though our results may be influenced by the outliers and subjective measurements of disease, there were robust correlations between three important clinical parameters. Prospective natural history studies to further validate the stratification of L-CMD based on ambulatory status, the special consideration of the association of the p.Arg249Trp *LMNA* variant with clinical severity, and a focus on outcome measures assessment between 5 and 15 years of age will improve clinical trial readiness and help in the design of future therapeutic clinical trials for this particular patient population with high medical need.

## Supplementary Material

fcab075_Supplementary_Data

## Abbreviations

CI =: confidence interval
DCM-CD =: isolated dilated cardiomyopathy with conduction system defects and arrhythmias
EDMD =: Emery – Dreifuss muscular dystrophy
ICD =: implantable cardioverter defibrillator
ICM =: Insertable Cardiac Monitor
IPPB =: intermittent positive pressure breathing
L-CMD =: *LMNA*-related congenital muscular dystrophy
LGMD1B =: limb-girdle muscular dystrophy type 1B
NIV =: non-invasive ventilation
