# Supplementary material for: International retrospective natural history study of LMNA-related congenital muscular dystrophy
Source: Brain Commun. 2021 Apr 11;3(3):fcab075. doi: 10.1093/braincomms/fcab075 (PMC8260964; doi:10.1093/braincomms/fcab075)

**Supplementary Table 1:** Numbers of patients according to neuromuscular referral centers where the patients were followed, age of first clinical manifestation and the referring clinicians.

| **Country (number of patients^+^)** | **City** | **Number of patients with first clinical manifestation <5 years old (190 patients)** | **Number of patients with first clinical manifestation <2 years old (151 patients)** | **Contributors** |
| --- | --- | --- | --- | --- |
| France  (n=35) | Garches | 17 | 15 | Quijano-Roy S; Dabaj I |
|  | Paris (AT*)  Paris (PS*) | 7  1 | 5  1 | Mayer M.  Voit T |
|  | Lille | 2 | 1 | Cuisset JM |
|  | Paris (Necker) | 2 | 2 | Desguerre I |
|  | Nantes | 2 | 0 | Mercier S |
|  | Strasbourg | 2 | 1 | Laugel V |
|  | Tours | 1 | 1 | Lagrue E |
|  | Reims | 1 | 1 | Sabouraud P |
| UK  (n=29) | London | 17 | 15 | Muntoni F; Sarkozy A |
|  | Newcastle | 12 | 9 | Bertoli M; Bushby K; Marini-Bettolo C |
| China (n=23) | Beijing | 23 | 22 | [Xiong H.](mailto:xh_bjbj@163.com) |
| Italy  (n=20) | Roma (IRCCS) | 5 | 5 | Mercuri E |
|  | Turin | 4 | 3 | Vercelli L |
|  | Milano | 3 | 2 | Maggi L |
|  | Naples | 3 | 3 | Politano L |
|  | Messina | 2 | 2 | Messina S |
|  | Roma (GCH) | 2 | 1 | D’Amico A |
|  | Pisa | 1 | 1 | Siciliano G |
| USA,  (n=19) | Torrance | 9 | 6 | Rutkowski A; R Alvarez (Cure CMD) |
|  | Bethesda | 9 | 8 | Bönnemann CG; Donkervoort S; Foley AR |
|  | Los Angeles | 1 | 1 | Pierson TM |
| Spain (n=17) | Barcelona | 17 | 7 | Nascimento A |
| Argentina (n=16) | Buenos Aires | 16 | 11 | Monges S |
| Brazil  (n=8) | Sao Paulo | 5 | 5 | Zanoteli E |
|  | Belo Horizonte | 3 | 3 | Gurgel Giannetti J |
| Japan (n=8) | Tokyo | 8 | 7 | Komali H; Ishiyama A |
| Chile  (n=5) | Sant. de Chile (CLC/RR*) | 2 | 2 | Kleinsteuber K |
|  | Santiago de Chile (LCM*) | 1 | 1 | Castiglioni C |
|  | Santiago de Chile (CLC*) | 2 | 2 | Erazo Torricelli R |
| Canada (n=3) | Toronto | 3 | 1 | Yoon G |
| Germany (n=3) | Essen | 3 | 3 | Schara U |
| Australia (n=2) | Victoria | 2 | 2 | Ryan M |
| Belgium (n=2) | Brussels | 2 | 2 | [Deconnick N](http://huderf.be/) |

+ Number of patients included at the initiation of the study, i.e. with first clinical manifestation <5 years old

* AT: Armand-Trousseau; PS: Pité-salpétrière; GCH: Gesù Children's Hospital; CLC: Clínica Las Condes; RR: Hospital Roberto del Río; LCM: Hospital Luis Calvo Mackenna;

**Supplementary Table 2:** Variables that were captured for each patient to populate the clinical data sheet:

| Demographic information: | Gender, date of birth, country of origin, referring center and clinician, country where attending neuromuscular clinic, date of last visit, date of death if applicable |
| --- | --- |
| *LMNA* mutation characteristics | DNA, amino acids modifications, Exon |
| Circumtunces of onset | Age and symptom(s) of onset |
| CK level | Maximal CK levels reached |
| Muscle biopsy main characteristics | Age at the biopsy, biopsied muscle, presence or absence of dystrophy, inflammation or other particular findings |
| Motor achievements | Ages of acquiring and losing of major motor milestones (Sitting, crawling, walking supported, walking unsupported and running) |
| Orthopedic features if applicable | Affected joints and age of onset of joint contractures and scoliosis, |
| Orthopedic interventions* if applicable | Age of joint contractures and scoliosis non-surgical and surgery treatments |
| Respiratory interventions* if applicable | Age of Intermittent positive pressure breathing, non-invasive ventilation, tracheostomy) |
| Cardiac abnormalities* if applicable | Age at onset of cardiac abnormalities (P-wave flattening, Arrhythmias, conduction defect, echocardiographic abnormalities) |
| Cardiac interventions* if applicable | Age of first heart specific drugs, pacemaker and implantable cardioverter defibrillator (ICD) |
| Corticosteroids usage if applicable | Age at starting and arrest, type of steroid |
| Gastrointestinal features and intervention* if applicable. | Age of any nutritional, feeding, metabolic abnormalities, gastrostomy feeding tube |

*****For the definition of cardiac abnormalities and cardiac, gastroenteric, orthopedic and respiratory interventions, see material and methods section.

**Supplementary Table 3:** *LMNA* variants identified in patients included in the study cohort. (according to *LMNA* # NM_170707.4 reference sequence).

| **Nucleotide change** | **Aminoacid change** | **Exon** | **Mutation type** | **Number of cases** |
| --- | --- | --- | --- | --- |
| c.91_93delGAG | p.Glu31del | exon 1 | small deletion (in frame) | 3 |
| c.91G>A | p.Glu31Lys | exon 1 | missense | 4 |
| c.94_96delAAG | p.Lys32del | exon 1 | small deletion (in frame) | 9 |
| c.94A>G | p.Lys32Glu | exon 1 | missense | 1 |
| c.96G>C | p.Lys32Asn | exon 1 | missense | 1 |
| c.103_105delCTG | p.Leu35del | exon 1 | small deletion (in frame) | 1 |
| c.104T>A | p.Leu35Gln | exon 1 | missense | 1 |
| c.104T>C | p.Leu35Pro | exon 1 | missense | 3 |
| c.109_111dup | p.Glu37dup | exon 1 | small insertion (in frame) | 1 |
| c.115A>G | p.Asn39Asp | exon 1 | missense | 1 |
| c.115A>T | p.Asn39Tyr | exon 1 | missense | 1 |
| c.116A>G | p.Asn39Ser | exon 1 | missense | 13 |
| c.117T>A | p.Asn39Lys | exon 1 | missense | 1 |
| c.117T>G | p.Asn39Lys | exon 1 | missense | 1 |
| c.121C>A | p.Arg41Ser | exon 1 | missense | 2 |
| c.122G>C | p.Arg41Pro | exon 1 | missense | 1 |
| c.125T>C | p.Leu42Ser | exon 1 | missense | 1 |
| c.128C>A | p.Ala43Glu | exon 1 | missense | 1 |
| c.143G>C | p.Arg48Pro | exon 1 | missense | 1 |
| c.149G>C | p.Arg50Pro | exon 1 | missense | 2 |
| c.305T>C | p.Leu102Pro | exon 1 | missense | 1 |
| c.392A>G | p.Gln131Pro | exon 2 | missense | 1 |
| c.422T>C | p.Leu141Pro | exon 2 | missense | 1 |
| c.513+2T>C | ? | intron 2 | intronic (splice site) | 1 |
| c.522_536del | p.175_179del | exon 3 | small deletion (in frame) | 1 |
| c.695G>A | p.Gly232Glu | exon 4 | missense | 1 |
| c.745C>T | p.Arg249Trp | exon 4 | missense | 30 |
| c.746G>A | p.Arg249Gln | exon 4 | missense | 3 |
| c.775T>A | p.Tyr259Asn | exon 4 | missense | 1 |
| c.781_783delAAG | p.261delLys | exon 4 | small deletion (in frame) | 1 |
| c.810+1G>C | ? | intron 4 | intronic (splice site) | 1 |
| c.832G>C | p.Ala278Pro | exon 5 | missense | 1 |
| c.854T>A | p.Val285Glu | exon 5 | missense | 1 |
| c.880_882delCAG | p.Gln294del | exon 5 | small deletion (in frame) | 1 |
| c.905T>C | p.Leu302Pro | exon 5 | missense | 1 |
| c.954G>A | p.Ala318Ala | exon 6 | missense | 1 |
| c.976T>A | p.Ser326Thr | exon 6 | missense | 1 |
| c.1072G>A | p.Glu358Lys | exon 6 | missense | 10 |
| c.1117A>G | p.Ile373Val | exon 6 | missense | 1 |
| c.1118T>A | p.Ile373Asn | exon 6 | missense | 1 |
| c.1124C>G | p.Ala375Gly | exon 6 | missense | 1 |
| c.1139T>C | p.Leu380Ser | exon 6 | missense | 1 |
| c.1147G>A | p.Glu383Lys | exon 6 | missense | 2 |
| c.1151A>G | p.Glu384Gly | exon 6 | missense | 1 |
| c.1163G>C | p.Arg388Pro | exon 7 | missense | 1 |
| c.1201C>T | p.Arg401Cys | exon 7 | missense | 1 |
| c.1325T>G | p.Val442Gly | exon 7 | missense | 1 |
| c.1337A>G | p.Asp446Gly | exon 7 | missense | 1 |
| c.1346G>T | p.Gly441Val | exon 7 | missense | 1 |
| c.1357C>T | p.Arg453Trp | exon 7 | missense | 7 |
| c.1358G>C | p.Arg453Pro | exon 7 | missense | 1 |
| c.1364G>C | p.Arg455Pro | exon 7 | missense | 1 |
| c.1366A>G | p.Asn456Asp | exon 7 | missense | 2 |
| c.1368C>A | p.Asn456Lys | exon 7 | missense | 1 |
| c.1368C>G | p.Asn456Lys | exon 7 | missense | 1 |
| c.1381-2A>G | ? | intron 7 | intronic (splice site) | 2 |
| c.1391T>G | p.Met464Arg | exon 8 | missense | 1 |
| c.1399T>A | p.Trp467Arg | exon 8 | missense | 1 |
| c.1478A>C | p.Gln493Pro | exon 8 | missense | 1 |
| c.1488_1488+9del | ? | exon 8 / intron8 | small intronic/exonic deletion | 1 |
| c.1488+1G>A | ? | intron 8 | intronic (splice site) | 1 |
| c.1489-14_1489-7del | ? | intron 8 | small intronic deletion | 1 |
| c.1540T>A | p.Trp514Arg | exon 9 | missense | 1 |
| c.1558T>C | p.Trp520Arg | exon 9 | missense | 1 |
| c.1580G>C | p.Arg527Pro | exon 9 | missense | 4 |
| c.1583C>G | p.Thr528Arg | exon 9 | missense | 2 |
| c.1583C>A | p.Thr528Lys | exon 9 | missense | 2 |

**Supplementary Table 4**. Outlier Analysis

| Patient ID | 26 | 60 | 79 | 183 | 186 |
| --- | --- | --- | --- | --- | --- |
| Country | France | England | Brazil | Italy | Italy |
| Age of Onset (years) | 2 | 0.5 | 2 | 2 | 2 |
| Max Motor Function | Run | Walk alone | Run | Walk alone | Walk alone |
| Age at Independent Ambulation (years) | 1.1 | 1.0 | 1.2 | 1.1 | 1.0 |
| Age at Walking Loss (years) | - | 30 | 38 | - | 36 |
| Age at 1^st^ Respiratory Intervention (years) | - | 29 | - | 10 | 35 |
| Age at 1^st^ Cardiac Abnormality (years) | 29.8 | - | 34 | 33 | 34 |
| Age at 1^st^ Cardiac Intervention (years) | 32.6 | 30 | - | 40 | 35 |

**Supplementary Figure 1:** Geographic distribution of the studied cohorts. Color indicated the number of case reported in the country.


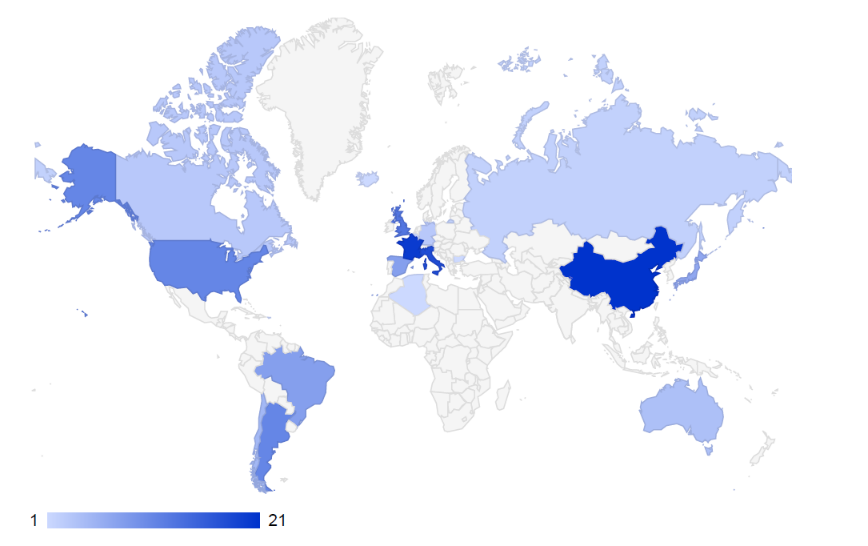

Supplement: fcab075_Supplementary_Data [file fcab075_supplementary_data.docx]
